# Mitochondrial haplogroups are not associated with diabetic retinopathy in a large Australian and British Caucasian sample

**DOI:** 10.1038/s41598-018-37388-8

**Published:** 2019-01-24

**Authors:** Ebony Liu, Georgia Kaidonis, Mark C. Gillies, Sotoodeh Abhary, Rohan W. Essex, John H. Chang, Bishwanath Pal, Mark Daniell, Stewart Lake, Jolly Gilhotra, Nikolai Petrovsky, Alex W. Hewitt, Alicia Jenkins, Ecosse L. Lamoureux, Jonathan M. Gleadle, Kathryn P. Burdon, Jamie E. Craig

**Affiliations:** 1 Department of Ophthalmology, Flinders University, Flinders Medical Centre, Adelaide, South Australia Australia; 2 Save Sight Institute, Clinical Ophthalmology and Eye Health, the University of Sydney, Sydney, New South Wales Australia; 3 Academic Unit of Ophthalmology, Australian National University, Canberra, Australia; 4 School of Medical Sciences, University of NSW, Sydney, New South Wales Australia; 5 Medical Retina Service, Moorfields Eye Hospital, London, United Kingdom; 6 Department of Ophthalmology, Royal Melbourne Hospital, Melbourne, Victoria Australia; 7 Department of Ophthalmology, Royal Adelaide Hospital, Adelaide, South Australia Australia; 8 Department of Endocrinology, Flinders University, Flinders Medical Centre, Adelaide, South Australia Australia; 9 Centre for Eye Research Australia, University of Melbourne, Melbourne, Victoria, Australia; 10 NHMRC Clinical Trials Centre, University of Sydney, Camperdown, New South Wales and St Vincent’s Hospital, Fitzroy, Victoria Australia; 11 Department of Renal Medicine, College of Medicine and Public Health, Flinders University, Adelaide, South Australia Australia; 12 Menzies Institute for Medical Research, University of Tasmania, Hobart, Tasmania Australia; 13 Duke-NUS Medical School, Singapore, Singapore

## Abstract

Mitochondrial haplogroups H1, H2 and UK have previously been reported to be associated with proliferative diabetic retinopathy (PDR) in Caucasian patients with diabetes. We aimed to replicate this finding with a larger sample and expand the analysis to include different severities of DR, and diabetic macular edema (DME). Caucasian participants (n = 2935) with either type 1 or type 2 diabetes from the Australian Registry of Advanced Diabetic Retinopathy were enrolled in this study. Twenty-two mitochondrial single nucleotide polymorphisms were genotyped by MassArray and haplogroups reconstructed using Haplogrep. Chi square tests and logistic regressions were used to test associations between haplogroup and DR phenotypes including any DR, non-proliferative DR (NPDR), proliferative DR (PDR) and DME. After stratifying the samples in type 1 and type 2 diabetes groups, and adjusting for sex, age, diabetes duration, concurrent HbA1c and hypertension, neither haplogroups H1, H2, UK, K or JT were associated with any DR, NPDR, PDR or DME.

## Introduction

Diabetic retinopathy (DR) is a leading cause of vision loss from diabetes driven damage to the retina. It is becoming increasingly prevalent in spite of better risk factor control and screening^1^. Globally, from 1990 to 2010, visual impairment attributable to diabetes increased by 64% from 2.3 million to 3.7 million^2^. Vision loss occurs from proliferative diabetic retinopathy (PDR) and diabetic macula edema (DME). PDR is the most severe form of DR and is characterized by the growth of pathological vessels in the retina. DME can occur at any stage of DR and is characterized by oedema in the macula region of the retina.

DR has a complex genetic component^3^. While several studies have explored genes involved in inflammation and angiogenesis related pathways (such as vascular endothelial growth factor), little research has focused on the role of mitochondrial DNA (mtDNA) in the susceptibility of DR^4,5^. It is well established that oxidative stress plays a key role in the pathogenesis of diabetic complications, including DR^6^. A significant source of reactive oxygen species (ROS) is from the mitochondria. Mitochondrial overproduction of ROS is hypothesized to be the single upstream event that mediates multiple mechanisms of hyperglycemia induced damage to tissues including polyol pathway flux, increased formation of advanced glycation end products (AGEs), increased expression of AGE receptors and activating ligands, activation of protein kinase C isoforms and overactivity of the hexosamine pathway^7^. Furthermore, mtDNA is highly sensitive to oxidative damage and has a high mutation rate with implications for electron transport chain function and endothelial cell survival, even long after the initial hyperglycemic insult^8–10^.

A common classification system for mtDNA variation is mitochondrial haplogroup, which represents the major branch points on the mitochondrial phylogenetic tree of human evolution. Estopinal *et al*. reported that haplogroups H1, H2 and UK in a Caucasian sample (n = 392) were associated with PDR^11^. Haplogroup H1 and H2 were risk factors for the development of PDR from non-proliferative diabetic retinopathy (NPDR), while haplogroup UK was protective against PDR. Subsequently, Bregman *et al*. reported similar findings in a larger group from the same population (n = 637), and reported further that while mitochondrial haplogroup was associated with PDR, it was not associated with DR more generally^12^. A different case control study (149 with any type of DR and 78 with no DR) found a higher prevalence of haplogroup T in those with any DR (12.1% vs 5.1%; p = 0.046)^13^.

We sought to replicate these studies in a larger sample (n = 2935) with increased power to explore other DR phenotypes such as DME and to evaluate this association in participants with type 1 and type 2 diabetes mellitus.

## Methods

### Ethics statement

This project has been approved by the human research ethics committees (HRECs) in Australia (Southern Adelaide Clinical HREC, Royal Adelaide Hospital HREC, The Queen Elizabeth Hospital HREC, Royal Melbourne Hospital HREC, Royal Victorian Eye and Ear Hospital HREC, St. Vincent’s Hospital (Melbourne) HREC, South Eastern Sydney Illawarra HREC, Tasmania Health and Medical HREC) and the NHS Health Research Authority in London. It adheres to the tenets of the Declaration of Helsinki. Written informed consent was obtained from each participant before study enrolment.

### Recruitment of patients and data collection

This study was carried out among Caucasian participants (identifying as of European descent) recruited in the Australian Registry of Advanced Diabetic Retinopathy (RADAR) and the Genetic Study of Diabetic Retinopathy based at Flinders University, South Australia. Multiple recruitment centres were involved and included the following Australian hospitals; Flinders Medical Centre, The Repatriation General Hospital, The Royal Adelaide Hospital, The Queen Elizabeth Hospital, The Royal Melbourne Hospital, Royal Victorian Eye and Ear Hospital, St. Vincent’s Hospital, Sydney Eye Hospital, Canberra Hospital, Royal Hobart Hospital, and from the United Kingdom; The National Institute for Health Research Biomedical Research Centre at Moorfields Eye Hospital NHS Foundation Trust and UCL Institute of Ophthalmology, London, United Kingdom.

Eligible participants were actively recruited from ophthalmology, diabetes and renal clinics, with the following inclusion criteria: 1) type 1 (T1DM) or type 2 diabetes mellitus (T2DM). Those with T2DM must have received at least 5 years of medical treatment for diabetes (oral hypoglycemic agents or insulin) prior to enrolment, and must have been over 18 years of age. All participants underwent a questionnaire and venous blood sample collection for DNA analysis. Clinical information was collected from case notes and electronic records, including the average of three most recent, available HbA1c measurements (or three measurements immediately prior to a diagnosis of PDR), renal and lipid measures, medications and the presence of non-ocular diabetic complications. DR grading (defined as the worst ever grading) and the presence of DME were determined from documented dilated fundus exams performed by an ophthalmologist. DR grading was defined by the International Clinical DR Severity Scale^14^. Clinically significant macula edema (CSME) was defined according to the Early Treatment Diabetic Retinopathy Study protocol: 1) retinal thickening within 500 μm of the center of the macula, 2) hard exudates at or within 500 μm of the centre of the macular if associated with thickening of the adjacent retina or 3) retinal thickening 1 disc area in size, within 1 disc diameter of the centre^15^. Sight threatening DR was defined as either severe NPDR, PDR or CSME.

For each participant, approximately 8 mL of blood was collected in EDTA blood collection tubes and underwent DNA extraction using the QIAamp Blood DNA Maxi Kits (Qiagen, Venlo, The Netherlands). More detail regarding the data collection method has been described previously^16^.

### Genotyping and mitochondrial haplogroup determination

Genotyping was performed through the Australian Genome Research Facility (AGRF), using the Agena Bioscience MassARRAY platform. We utilized the same panel of 22 mtDNA SNPs designed by Estopinal *et al*. in previous studies to determine mitochondrial haplogroup (Supplementary Table 1)^11^. Haplogrep software was used to facilitate haplogroup identification^17^. Samples identified as non-Caucasian after haplogroup determination were removed from the analyses.

### Statistical analyses

Statistical analysis was performed with Statistical Package for Social Sciences versions 23.0 (For Windows; IBM Corp, Armonk, NY). Chi Square tests were performed to analyse the association of haplogroup type with various DR phenotypes such as any DR, any NPDR, PDR, DME and CSME. Logistic regression was used to adjust for covariates age, sex, type of diabetes, duration of diabetes, HbA1c and presence of hypertension. Statistical significance was taken at p < 0.05. Further analysis was performed by stratifying the analysis into T1DM and T2DM cohorts, and the major European haplogroups (H1 and H2, UK).

## Results

Patient demographics (n = 2935) stratified by DR phenotype are presented in Table 1. Chi square tests and Mann-Whitney U tests were used to compare demographic variables between the different phenotype groups (Table 2). Diabetes duration, HbA1c and hypertension were associated with all subtypes of DR and DME. Diabetes duration and HbA1c significantly increased from no DR to NPDR, PDR, DME or CSME(p < 0.0001, Mann-Whitney U test) and similarly from NPDR to PDR. Type of diabetes was also a significant variable affecting DR phenotypes PDR, any DME and CSME. Participants with PDR were younger than those with NPDR (median 59 versus 64 years, p < 0.0001).

**Table 1:** Demographics of study population stratified by diabetic retinopathy phenotype.

Demographic	Total	No DR	Any DR	Any NPDR	PDR	Any DME	CSME	Sight threatening
n	2935	1124	1811	1161	650	936	643	1278
Female; n (%)	1309 (44.6)	521 (46.5)	788 (43.7)	514 (44.4)	274 (42.4)	420 (45.5)	289 (45.2)	558 (43.9)
Age in yrs; median (range)	61 (17–95)	65 (17–95)	63 (18–95)	65 (18–95)	59 (21–90)	65 (21–92)	65 (26–92)	63 (21–92)
Diabetes duration in yrs; median (range)	18 (5–70)	12 (5–67)	20 (5–70)	18 (5–65)	23 (5–70)	19 (5–64)	19 (5–59)	20 (5–70)
Type 1 diabetes; n (%)	670 (22.8)	239 (21.5)	431 (24.1)	208 (18.2)	223 (34.6)	148 (16.0)	100 (15.7)	297 (23.5)
HbA1c %; median (range)	8.07 (2–22)	7.40 (2–22)	8.10 (4–15)	7.90 (5–15)	8.50 (4–15)	8.20 (4–15)	8.10 (5–15)	8.25 (4–15)
Hypertension; n (%)	1935 (65.9)	695 (61.8)	1240 (68.5)	782 (67.4)	458 (70.5)	642 (68.6)	471 (73.3)	874 (68.4)

**Table 2:** P values of demographic variables compared between the DR phenotype groups.

	No DR vs any DR	No DR vs any NPDR	No DR vs PDR	No DR vs DME	No DR vs CSME	No DR vs Sight threatening
Sex	0.077	0.333	0.102	0.563	0.619	0.216
Age	0.103	0.190	**<0**.**0001**	0.761	0.848	**0**.**041**
Diabetes duration	**<0**.**0001**	**<0**.**0001**	**<0**.**0001**	**<0**.**0001**	**<0**.**0001**	**<0**.**0001**
Diabetes type	0.103	0.051	**<0**.**0001**	**1**.**79** × **10**^**−3**^	**3**.**79** × **10**^**−3**^	0.258
HbA1c	**<0**.**0001**	**<0**.**0001**	**<0**.**0001**	**<0**.**0001**	**<0**.**0001**	**<0**.**0001**
Hypertension	**3.74 ×** **10**^**−4**^	**2.91 × 10** ^**−3**^	**1.18 × 10** ^**−3**^	**2.36 × 10** ^**−3**^	**<0**.**0001**	**1.37 × 10** ^**−3**^

### Mitochondria haplogroup and DR phenotype

A total of 7 European mitochondrial haplogroups were identified in our Caucasian sample. The most common ones were haplogroup H1 and H2 (analysed collectively) and UK at 50.8% and 22.5% respectively. Other types included JT (12.4%), R (7.1%), I (4.2%), W (2.0%) and X (1.0%) (Supplementary Table 2). One SNP (rs3088053, rCRS position 11812) failed genotyping and therefore Haplogroup T2 could not be identified in our samples. As T2 is a subtype of J, we have therefore combined haplogroups J, T1 (and T2) in our analyses.

We found the percentages of the three most common haplotype groups (H1 and H2, UK and JT) were distributed similarly in each of the different phenotype groups, and that any differences when compared with no DR controls were not statistically significant after performing Chi Square association tests (Table 3). We also found no significant associations when haplogroups were compared between NPDR and PDR. There were no significant differences when all 7 haplogroups were analysed separately instead of grouping less common haplogroups into one category.

**Table 3:** Haplogroup distribution (H, UK, JT, Other) according to DR phenotype.

Haplogroup	Proportion (%)				P value (Chi Square tests) comparison to controls (No DR)
	H	UK	JT	I, R,W,X	
No DR	50.7	22.5	12.2	14.6	NA
Any DR	50.4	22.9	12.9	13.8	0.885
NPDR	50.9	22.9	12.4	13.8	0.954
PDR	49.5	22.8	13.8	13.8	0.760
DME	51.0	23.3	12.5	13.2	0.830
CSME	49.6	23.3	13.2	13.8	0.867
Sight threatening DR	50.8	23.2	12.9	13.1	0.721
NPDR	50.4	22.9	12.8	13.9	0.793 (compared with PDR)

After separating the samples per diabetes type, the majority were T2DM participants (n = 2265) compared with T1DM participants (n = 670). The demographics of the T1DM and T2DM groups are given in Supplementary Tables 3 and 5 respectively. P values comparing the demographic variables between cases and controls in the T1DM and T2DM groups are given in Supplementary Table 5 and 6 respectively. Duration of diabetes, HbA1c and the presence of hypertension were significantly increased from no DR to NPDR, PDR, DME or CSME (p < 0.01, Mann-Whitney U test) and similarly from NPDR to PDR in both types of diabetes. Binary logistic regression show that haplogroups H1 and H2, and UK were not associated with any DR phenotypes in either T1DM or T2DM after adjustment for sex, age, diabetes duration, HbA1c and hypertension (Tables 4 and 5). After logistic regression, diabetes duration and HbA1c remain significant risk factors for DR in both type 1 and type 2 diabetes, while hypertension only remained significant in type 1 diabetes.

**Table 4:** Association of haplogroup H (H1 and H2) and UK with DR in type 1 diabetes: binary logistic regression adjusting for gender, age, diabetes duration and HbA1c.

OR (95% CI) P-value	No DR vs Any DR	No DR vs NPDR	No DR vs PDR	No DR vs DME	No DR vs CSME	No DR vs sight threatening	NPDR vs PDR
**Haplogroup H – Type 1 Diabetes**							
Haplogroup H	0.90 (0.58–1.40) P = 0.652	0.92 (0.57–1.48) P = 0.718	0.95 (0.53–1.69) P = 0.848	0.95 (0.52–1.77) P = 0.881	0.79 (0.38–1.63) P = 0.524	0.97 (0.58–1.63) P = 0.918	0.98 (0.62–1.55) P = 0.924
Sex (female)	1.34 (0.86–2.09) P = 0.199	1.30 (0.80–2.10) P = 0.294	1.44 (0.79–2.62) P = 0.231	1.43 (0.77–2.64) P = 0.255	1.54 (0.78–3.17) P = 0.243	1.43 (0.85–2.41) P = 0.182	0.84 (0.53–1.33) P = 0.453
Age	1.02 (1.0–1.03) P = 0.063	1.02 (1.0–1.04) **P** = **0.011**	0.99 (0.96–1.01) P = 0.347	1.04 (1.02–1.06) **P** = **3.81 **× **10**^**−3**^	1.03 (1.01–1.06) **P** = **0.007**	1.02(1.0–1.04) P = 0.104	0.97 (0.95–0.99) **P** = **0.001**
Diabetes duration	1.13 (1.10–1.16) **P** < **0.0001**	1.10 (1.07–1.13) **P** < **0.0001**	1.14 (1.11–1.19) **P** < **0.0001**	1.08 (1.04–1.11) **P** < **0.0001**	1.08 (1.04–1.12) **P** < **0.0001**	1.13 (1.10–1.17) **P** < **0.0001**	1.08 (1.06–1.11) **P** < **0.0001**
HbA1c	1.46 (1.26–1.70) **P** < **0.0001**	1.38 (1.18–1.63) **P** < **0.0001**	1.54 (1.28–1.86) **P** < **0.0001**	1.57 (1.30–1.91) **P** < **0.0001**	1.49 (1.19–1.87) **P** < **0.0001**	1.52 (1.28–1.79) **P** < **0.0001**	1.25 (1.07–1.46) **P** = **0.004**
Hypertension	2.19 (1.30–3.68) **P** < **0.0001**	1.61 (0.90–2.87) P = 0.108	4.34 (2.24–8.41) **P** < **0.0001**	2.60 (1.34–5.07) **P** = **4.85 **× **10**^**−3**^	2.61 (1.19–5.73) **P** = **0.017**	2.82 (1.57–5.05) **P** = **0.001**	2.21 (1.31–3.73) **P** = **0.003**
**Haplogroup UK – Type 1 Diabetes**							
Haplogroup UK	0.80 (0.49–1.32) P = 0.391	0.68 (0.39–1.21) P = 0.191	(0.53–1.91) P = 0.989	0.65 (0.31–1.36) P = 0.255	0.50 (0.21–1.24) P = 0.136	0.88 (0.49–1.58) P = 0.674	1.60 (0.93–2.75) P = 0.087
Sex (female)	1.34 (0.86–2.09) P = 0.201	1.28 (0.79–2.08) P = 0.311	1.44 (0.79–2.62) P = 0.231	1.46 (0.79–2.70) P = 0.231	1.53 (0.74–3.16) P = 0.251	1.43 (0.85–2.42) P = 0.177	0.84 (0.53–1.34) P = 0.457
Age	1.02 (1.0–1.03) P = 0.064	1.02 (1.00–1.04) **P** = **0.011**	0.99 (0.96–1.01) P = 0.351	1.04 (1.02–1.06) **P** = **4.17** × **10**^**−4**^	1.04 (1.00–1.06) **P** = **0.007**	1.02 (1.00–1.04) P = 0.108	0.97 (0.95–0.99) **P** = **0.001**
Diabetes duration	1.13 (1.09–1.16) **P** < **0.0001**	1.10 (1.06–1.13) **P** < **0.0001**	1.14 (1.11–1.19) **P** < **0.0001**	1.08 (1.04–1.11) **P** < **0.0001**	1.08 (1.04–1.12) **P** < **0.0001**	1.13 (1.10–1.17) **P** < **0.0001**	1.08 (1.06–1.11) **P** < **0.0001**
HbA1c	1.46 (1.26–1.70) **P** < **0.0001**	1.39 (1.18–1.63) **P** < **0.0001**	1.54 (1.28–1.86) **P** < **0.0001**	1.56 (1.28–1.89) **P** < **0.0001**	1.47 (1.17–1.85) **P** = **0.001**	1.51 (1.28–1.79) **P** < **0.0001**	1.25 (1.07–1.46) **P** = **0.004**
Hypertension	2.22 (1.32–3.75) **P** = **0.003**	1.67 (0.93–2.98) P = 0.086	4.34 (2.23–8.42) **P** < **0.0001**	2.75 (1.40–5.41) **P** = **0.003**	2.86 (1.28–6.40) **P** = **0.011**	2.85 (1.58–5.13) **P** = **4.80** × **10**^**−4**^	2.27 (1.35–3.84) **P** = **0.002**

**Table 5:** Association of haplogroup H and UK with DR in type 2 diabetes: binary logistic regression adjusting for gender, age, diabetes duration and HbA1c.

OR, 95% CI, P value	No DR vs Any DR	No DR vs NPDR	No DR vs PDR	No DR vs DME	No DR vs CSME	No DR vs sight threatening	NPDR vs PDR
**Haplogroup H – Type 2 Diabetes**							
Haplogroup H	1.03 (0.83–1.28) P = 0.777	1.07 (0.85–1.34) P = 0.571	0.92 (0.66–1.27) P = 0.612	1.04 (0.80–1.34) P = 0.780	0.91 (0.69–1.21) P = 0.521	1.04 (0.81–1.32) P = 0.763	0.93 (0.69–1.23) P = 0.598
Sex (female)	0.75 (0.60–0.93) **P** **=** **0**.**008**	0.76 (0.60–0.96) **P** **=** **0**.**019**	0.70 (0.50–0.97) **P** **=** **0**.**033**	0.87 (0.67–1.12) P = 0.270	0.87 (0.65–1.15) P = 0.328	0.79 (0.63–1.01) P = 0.064	0.95 (0.70–1.27) P = 0.711
Age	0.98 (0.97–0.99) **P** < **0**.**0001**	0.99 (0.98–1.0) **P** = **0**.**016**	0.96 (0.94–0.97) **P** < **0**.**0001**	0.98 (0.97–0.99) **P** = **4**.**81** × **10**^**−4**^	0.98 (0.97–0.99) **P** = **0**.**002**	0.97 (0.96–0.99) **P** < **0**.**0001**	0.97 (0.96–0.98) **P** < **0**.**0001**
Diabetes duration	1.09 (1.08–1.11) **P** < **0**.**0001**	1.08 (1.07–1.10) **P** < **0**.**0001**	1.12 (1.10–1.15) **P** < **0**.**0001**	1.10 (1.08–1.12) **P** < **0**.**0001**	1.10 (1.08–1.12) **P** < **0**.**0001**	1.11 (1.09–1.12) **P** < **0**.**0001**	1.04 (1.02–1.06) **P** < **0**.**0001**
HbA1c	1.37 (1.27–1.48) **P** < **0**.**0001**	1.33 (1.22–1.44) **P** < **0**.**0001**	1.50 (1.34–1.67) **P** < **0**.**0001**	1.45 (1.32–1.58) **P** < **0**.**0001**	1.44 (1.30–1.60) **P** < **0**.**0001**	1.45 (1.33–1.58) **P** < **0**.**0001**	1.15 (1.06–1.26) **P** = **0**.**001**
Hypertension	0.96 (0.74–1.24) P = 0.742	0.94 (0.71–1.25) P = 0.681	0.96 (0.65–1.43) P = 0.846	0.77 (0.57–1.04) P = 0.092	1.06 (0.74–1.51) P = 0.758	0.83 (0.62–1.11) P = 0.208	1.01 (0.71–1.44) P = 0.943
**Haplogroup UK – Type 2 Diabetes**							
Haplogroup UK	(0.77–1.29) P = 0.981	0.98 (0.74–1.29) P = 0.863	1.13 (0.76–1.67) P = 0.541	1.08 (0.80–1.46) P = 0.630	1.13 (0.81–1.59) P = 0.464	1.03 (0.77–1.38) P = 0.849	1.07 (0.75–1.51) P = 0.714
Sex (female)	0.75 (0.60–0.93) **P** = **0**.**008**	0.76 (0.60–0.95) **P** = **0**.**018**	0.70 (0.50–0.97) **P** = **0**.**034**	0.87 (0.67–1.12) P = 0.267	0.87 (0.66–1.16) P = 0.348	0.79 (0.62–1.01) P = 0.063	0.95 (0.70–1.27) P = 0.712
Age	0.98 (0.97–0.99) **P** < **0**.**0001**	0.99 (0.98–1.0) **P** = **0**.**016**	0.96 (0.94–0.97) **P** < **0**.**0001**	0.98 (0.97–0.99) **P** = **4**.**73** × **10**^**−4**^	0.98 (0.97–0.99) **P** = **0**.**002**	0.97 (0.96–0.99) **P** < **0**.**0001**	0.97 (0.96–0.98) **P** < **0**.**0001**
Diabetes duration	1.09 (1.08–1.11) **P** < **0**.**0001**	1.08 (1.07–1.10) **P** < **0**.**0001**	1.12 (1.10–1.15) **P** < **0**.**0001**	1.10 (1.08–1.12) **P** < **0**.**0001**	1.10 (1.08–1.12) **P** < **0**.**0001**	1.11 (1.09–1.12) **P** < **0**.**0001**	1.04 (1.02–1.06) **P** < **0**.**0001**
HbA1c	1.37 (1.27–1.48) **P** < **0**.**0001**	1.33 (1.22–1.44) **P** < **0**.**0001**	1.50 (1.34–1.67) **P** < **0**.**0001**	1.45 (1.32–1.58) **P** < **0**.**0001**	1.44 (1.30–1.60) **P** < **0**.**0001**	1.45 (1.33–1.58) **P** < **0**.**0001**	1.15 (1.06–1.26) **P** = **0**.**001**
Hypertension	0.96 (0.74–1.25) P = 0.746	0.95 (0.72–1.25) P = 0.695	0.96 (0.64–1.43) P = 0.839	0.77 (0.57–1.04) P = 0.091	1.05 (0.74–1.50) P = 0.772	0.83 (0.62–1.11) P = 0.207	1.01 (0.71–1.44) P = 0.953

The next most common haplogroups (JT and K separately from UK) were analysed separately (frequencies 12.4% and 7.8% respectively). Significant results were: haplogroup K was nominally associated with any DR (135 cases, 98 controls, OR 0.49, 96% CI 0.24–1.00, p = 0.05) and NPDR (85 cases, 98 controls, OR 0.31, 95% CI 0.13–0.78, p = 0.012). JT was nominally associated with NPDR (144 cases, 137 controls, OR 2.20, 95%CI 1.09–4.43, p = 0.027) and CSME (85 cases, 137 controls, OR 2.06, 95% CI 1.16–8.08, p = 0.024) These results should be treated with caution as the numbers are small and the association does not survive correction for multiple hypothesis testing.

## Discussion

In our larger Caucasian sample, unlike earlier smaller studies, we found no significant associations between mitochondrial haplogroup and the presence of any DR, DME, nor more severe phenotypes such as PDR, CSME or sight-threatening DR (severe NPDR, CSME or PDR). This was true for analysis as a group or when stratified for type of diabetes, in spite of following the same methods of previous studies which found positive associations.

Estopinal *et al*. first demonstrated that haplogroups H1 and H2 (analysed collectively) and UK were associated with PDR when compared with NPDR in an American Caucasian sample (n = 197 NPDR, 195 PDR)^11^. Having either haplogroup H1 or H2 was a risk factor, while haplogroup UK was protective. Bregman *et al*. expanded from this initial study with 513 additional diabetic controls in the same databases (Vanderbilt Eye Institute and Vanderbilt University)^12^. They found that haplogroup H1 and H2, and UK were not associated with any incident DR compared with no DR. Using the same cohort, Mitchell *et al*. found duration of diabetes and HbA1c was significantly associated with PDR in haplogroups H1 and H2, but not UK, suggesting that mitochondrial haplogroups modify these clinical risk factors for the development of PDR in type 2 diabetes^18^. In a different study, Kofler *et al*. reported haplogroup T was significantly associated with any DR compared with no DR (12.1% vs 5.1%; p = 0.046)^13^.

Inconsistent results are common in all areas of haplogroup association studies. For example Crispim *et al*. reported haplogroup cluster J/T was significantly associated with insulin resistance in a Caucasian Brazilian population^19^, but this was refuted by two other studies of Caucasian samples^20,21^. Challenges in interpreting mitochondrial association studies include differences in study design, case and control definitions, statistical analysis, population stratification, inadequate power and lack of replication^22^.

Different results could be due to different populations and study design, however in examining the demographics and distribution of the haplogroups, our group appears to be similar to the group from the Vanderbilt Eye Institute and Vanderbilt University. Both groups consist of Caucasian patients of European descent. We used the same criteria for selection of retinopathy cases and controls and the same statistical analyses. The most common haplogroups were H1 and H2, and UK; 73.3% in our study, compared with 68% in Bregman *et al*.'s study. As expected in both studies, age, diabetes duration, type of diabetes and HbA1c were strongly associated with increasing severity of DR.

An important reason why our results are different is because our study consisted of a much larger population; 1124 diabetic retinopathy controls (no DR), 1161 NPDR cases and 650 PDR cases. Therefore our study has increased statistical power to identify any true associations. We were unable to replicate previously reported associations, suggesting that these previous association may be false. Smaller studies and sub analyses of phenotype groups lead to a higher risk of type 1 errors^23^. Our larger study size allowed us to analyse other phenotypes such as DME and CSME, as well as to separately analyse less common haplogroups such as JT, and K separate from UK. The only statistically significant results we found were haplogroup K was nominally associated with any DR, and haplogroup JT was nominally associated with NPDR and CSME. As the numbers were small in these comparisons and the result does not survive multiple hypothesis testing, this is likely to be type 1 error. Haplogroup K was not a common haplogroup in the previous two studies and is not implicated in diabetes and other associated diseases. Kofler *et al*. reported haplogroup T was significantly associated with any DR but this study also had a much smaller sample size (149 with any DR and 78 with no DR)^13^. As noted in our results, we were unable to separately analyse haplogroup T due to genotyping failure, and so direct comparison to Kofler *et al*.’s study could not be made.

In addition to study size, strengths of this study include the inclusion of both T1DM and T2DM subjects from multiple sites, rigour of retinopathy status characterisation, wide range of levels of DR and use of the same haplotyping methods and statistical analyses as previous studies so comparisons could be made.

The haplotyping method we utilized from previous studies has an important limitation. SNPs chosen to represent the H haplogroup (rCRS position 3010 and 1438) only identify haplogroups H1 and H2. Therefore 7 other major subtypes of haplogroup H were not analysed. In our study, one SNP completely failed genotyping (rCRS 11812, determination of haplogroup T2) and therefore we could not analyse haplogroup T separately. Another 4 SNPs had a 2% failure rate, and this could have contributed to the percentage of samples with haplogroup R (a major clade consisting of H, J, T, and UK). We chose our Caucasian sample based on participants self-identifying as Caucasian, but a small number had non-Caucasian haplogroups (for example haplogroup A, B, C, L, M, N and Q). Some of the 22 SNPs chosen for haplogroup determination are also found in other ethnic populations (for example rCRS position 3197 determines U5 but also L3e3 which is found in Asian populations). Therefore, all samples with a non-Caucasian haplogroup were removed to minimize any confounding effect and reduce population stratification.

We recognise that even larger studies and studies in different ethnic groups, particularly those at high risk of diabetic retinopathy, are desirable. We only studied 7 haplogroups, while the human mitochondrial phylogenetic tree consists of hundreds of haplogroups. The hypothesis that variations in mitochondrial genetics contribute to DR risk is logical given the role that the mitochondria play in oxidative stress^7^ and their presence in the retina^24^. Mitochondrial DNA are inherited completely from the maternal line, unlike nuclear DNA which has equal maternal and paternal contributions^25^. Risk of T1DM in the offspring varies by parental status; being two-fold lower if the mother has T1DM rather than the father^26^. Epidemiology studies show that certain ethnicities are at greater risk of DR such as people of Asian, African and Indigenous ethnic groups^27^. Complex biological and environmental factors explain this observation, and mitochondrial genetics could also play a role, but this has not been studied.

Mitochondrial haplogroup is not a specific marker for mitochondrial genetic variability. A haplogroup consists of many genetic variants that are inherited together. Therefore, if there are specific mitochondrial variants that contribute to DR, these cannot be studied effectively. Single nucleotide polymorphisms in mitochondria cause diseases such as Leber hereditary optic neuropathy, and in complex diseases such as diabetes and cancer, it is increasingly recognised that small mitochondrial defects could lead to subtle bioenergetics alterations with major clinical implications^28^. Specific mitochondrial variants that have been studied for DR include mutations in *UCP2* and *Mn-SOD* genes^4^.

Few studies have demonstrated whether mitochondrial haplogroup directly affects mitochondrial function. Fang *et al*. recently reported lower respiratory chain complex activity in haplogroup N9a compared with D4j, G4a2 and Y1, using transmitochondrial technology^29^. Mueller *et al*. reported mitochondrial haplogroup T cell cybrids had a higher survival rate than haplogroup H cybrids under oxidative stress conditions such as when challenged with hydrogen peroxide^30^. Haplogroup K cybrids showed different gene expression levels compared with H cybrids after amyloid-beta toxicity^31^. Untreated retinal cell cybrids of H and J haplogroups also showed different gene expression and methylation status^32^. Future studies and techniques designed to explore the mitochondria genome in better detail than currently available can help us understand whether mitochondrial genetics contribute to DR risk^33^.

## Conclusion

In contrast to previous studies, our much larger study found no association between the major European mitochondrial haplogroup H1, H2, UK, and DR phenotypes in either type 1 or type 2 diabetes. No significant associations were found for different severities of DR and DME, or other subsets of mitochondrial haplogroups that were analysed by this study.

## Supplementary information

Supplementary Tables

## Data Availability

The dataset generated for analysis in the current study is available under a CC BY-NC-ND license at: 10.25957/5c060cbdb9162.
