# Supplementary material for: Mitochondrial haplogroups are not associated with diabetic retinopathy in a large Australian and British Caucasian sample
Source: Sci Rep. 2019 Jan 24;9:612. doi: 10.1038/s41598-018-37388-8 (PMC6345891; doi:10.1038/s41598-018-37388-8)
Supplement: Supplementary file 1 — Supplementary Tables [file 41598_2018_37388_MOESM1_ESM.pdf]

Supplementary Tables

Title: Mitochondrial haplogroups are not associated with diabetic retinopathy in a large Australian and British Caucasian sample

Authors: *Ebony Liu[1] MBBS, Georgia Kaidonis[1] MBBS, PhD, Mark C Gillies[2] FRANZCO, PhD, Sotoodeh Abhary[1] MBBS, PhD, Rohan W Essex[3] FRANZCO, John H Chang[4,5] FRANZCO, PhD, Bishwanath Pal[5] FRCO, Mark Daniell[6] FRANZCO, Stewart Lake[1] FRANZCO, Jolly Gilhotra[7] M.Med, FRANZCO, Nikolai Petrovsky[8] MBBS, PhD, Alex W Hewitt[9] FRANZCO, PhD, Alicia Jenkins[10] FRACP, PhD, Ecosse L Lamoureux[9,13] PhD, Jonathan M Gleadle[11] DPhil, FRACP, #Kathryn P Burdon[1,12]PhD, #Jamie E Craig[1] FRANZCO, DPhil. #Contributed equally

Supplementary table 1: List of 22 SNPs used for haplogroup determination [11]

SNP ID	rCRS position	Haplogroup
rs2001030	1438	H2
rs28358576	1811	U
rs3928306	3010	J1, H1
rs2854131	3197	U5
rs2854134	3594	L0-2
rs28357980	4917	T
rs3021088	5460	W
rs41347846	10034	I
rs28358275	10238	I
rs2857284	10873	N
rs2853493	11467	U
rs3088053	11812	T2
rs28359168	11947	W
rs2853498	12308	U
rs2853499	12372	U
rs3926883	12633	T1
rs2854122	12705	R
rs2853503	13617	U5
rs28359178	13708	J
rs3135030	14470	X
rs28357681	14798	UK, J1c
rs41518645	15257	J2

Supplementary table 2: Haplogroup frequencies in this study

Haplogroup	n (%)
H	1483 (50.5)
UK	667 (22.7)
JT	371 (12.6)
R	208 (7.1)
I	122 (4.2)
W	57 (1.9)
X	26 (0.9)

Supplement table 3: Demographics of type 1 diabetic group

Demographic	No DR	Any DR	Any NPDR	PDR	Any DME	CSME	Sight threatening
n	239	431	208	223	148	100	297
Female (n, %)	106 (44.5)	212 (49.3)	95 (45.7)	104 (46.8)	80 (54.1)	55 (55.0)	145 (49.0)
Age, yrs (median, range)	31 (17-83)	48 (18-88)	48 (18-88)	48 (21-85)	52 (21-88)	52 (26-85)	49 (21-88)
Diabetes duration, yrs (median, range)	13 (5-60)	27 (6-70)	23 (6-65)	31 (8-70)	26 (7-64)	27 (8-59)	29 (7-70)
HbA1c % (median, range)	7.8 (5-15)	8.4 (5-14)	8.2 (5-14)	8.7 (5-15)	8.0 (5-15)	8.6 (5-14)	8.6 (5-14)
Hypertension (n, %)	58 (24.3)	239 (55.5)	95 (45.7)	144 (64.6)	86 (58.1)	59 (59.0)	179 (60.3)

Supplementary table 4: P values of demographic variables compared between the DR phenotype groups in type 1 diabetes

	No DR vs any DR	No DR vs any NPDR	No DR vs PDR	No DR vs DME	No DR vs CSME	No DR vs Sight threatening
Female	0.258	0.129	0.640	0.075	0.095	0.337
Age	**<0.0001**	**<0.0001**	**<0.0001**	**<0.0001**	**<0.0001**	**<0.0001**
Diabetes duration	**<0.0001**	**<0.0001**	**<0.0001**	**<0.0001**	**<0.0001**	**<0.0001**
HbA1c	**<0.0001**	**0.008**	**<0.0001**	**<0.0001**	**0.007**	**<0.0001**
Hypertension	**<0.0001**	**<0.0001**	**<0.0001**	**<0.0001**	**<0.0001**	**<0.0001**

Supplementary table 5: Demographics of type 2 diabetic group

Demographic	No DR	Any DR	Any NPDR	PDR	Any DME	CSME	Sight threatening
n	875	1359	938	421	775	535	966
Female (n, %)	408 (46.9)	565 (41.7)	395 (42.2)	170 (40.5)	332 (43.0)	230 (43.2)	405 (42.1)
Age, yrs (median, range)	68 (24-95)	66 (27-95)	67 (27-95)	63 (31-90)	65 (31-92)	65 (31-92)	65 (31-92)
Diabetes duration, yrs (median, range)	11 (5-67)	17 (5-58)	17 (5-58)	20 (5-55)	18 (5-58)	18 (5-58)	18 (5-58)
HbA1c % (median, range)	7.3 (2-22)	7.9 (4-15)	7.8 (5-15)	8.4 (4-15)	8.1 (4-15)	8.1 (5-15)	8.1 (4-15)
Hypertension (n, %)	636 (77.5)	995 (77.8)	684 (78.0)	311 (77.4)	553 (75.6)	409 (80.0)	691 (76.0)

Supplementary 6: P values of demographic variables compared between the DR phenotype groups in type 2 diabetes

	No DR vs any DR	No DR vs any NPDR	No DR vs PDR	No DR vs DME	No DR vs CSME	No DR vs Sight threatening
Female	**0.016**	**0.047**	**0.032**	0.123	0.185	**0.038**
Age	**<0.0001**	**0.047**	**<0.0001**	**<0.0001**	**<0.0001**	**<0.0001**
Diabetes duration	**<0.0001**	**<0.0001**	**<0.0001**	**<0.0001**	**<0.0001**	**<0.0001**
HbA1c	**<0.0001**	**<0.0001**	**<0.0001**	**<0.0001**	**<0.0001**	**<0.0001**
Hypertension	0.872	0.816	1.00	0.400	0.274	0.494
